# Molecular epidemiology and clinical significance of carbapenemase genes in carbapenem-resistant *Acinetobacter baumannii* isolates in southern Poland

**DOI:** 10.20452/pamw.16734

**Published:** 2024-04-23

**Authors:** Piotr A. Serwacki, Dariusz A. Hareza, Anna Kujawska, Anna Pałka, Estera Jachowicz-Matczak, Agata Rybka-Grymek, Wioletta Świątek-Kwapniewska, Iwona Pawłowska, Zofia Gniadek, Karolina Gutkowska, Mateusz Gajda, Jadwiga Wójkowska-Mach

**Affiliations:** 1 Department of Anesthesiology and Intensive Care, St. Luke’s Provincial Hospital, Tarnów, Poland; 2 Department of Medicine, Johns Hopkins University School of Medicine, Baltimore, Maryland, United States; 3 Department of Microbiology, University Hospital in Krakow, Kraków, Poland; 4 Department of Microbiology, Faculty of Medicine, Jagiellonian University Medical College, Kraków, Poland; 5 Laboratory of Microbiology and Epidemiology, District Hospital in Bochnia, Bochnia, Poland; 6 Laboratory of Microbiology, St. Luke’s Provincial Hospital, Tarnów, Poland; 7 Division of Microbiology, St. Barbara Specialized Regional Hospital No. 5, Sosnowiec, Poland

**Keywords:** *Acinetobacter baumannii*, antimicrobial resistance, oxacillinases, Poland

## Abstract

**INTRODUCTION:**

A complex interplay between *Acinetobacter* spp., patients, and the environment has made it increasingly difficult to optimally treat patients infected with *Acinetobacter* spp., mainly due to rising antimicrobial resistance and challenges with surveillance.

**OBJECTIVES:**

This study evaluated carbapenem-resistant *A*. *baumannii* (CRAB) isolates to determine their resistance profiles and the presence of specific β-lactamases to inform CRAB surveillance upon hospital admission and regional empiric antibiotic therapies.

**PATIENTS AND METHODS:**

The study was conducted at 4 hospitals in southern Poland between June and December 2022. Only health care–associated infections caused by *A*. *baumannii* were considered. A total of 82 CRAB isolates were included in the analysis. Species identification was performed by matrix-assisted laser desorption / ionization time-of-flight mass spectrometry, antimicrobial susceptibility was determined phenotypically, and polymerase chain reactions were carried out to identify the resistance genes.

**RESULTS:**

Depending on the hospital, the incidence of CRAB infections varied from 428.6 to 759.5 per 10 000 admissions in intensive care units (ICUs), and from 0.3 to 21 per 10 000 admissions in non-ICUs. CRAB antibiotic susceptibility was the highest for cefiderocol (100%), colistin (96%), tigecycline (77%), gentamicin (51%), and ampicillin / sulbactam (36%). The most prevalent *bla*_OXA_ genes were *bla*_OXA-66–1_ (95%) and *bla*_OXA-40_ (71%), and additionally the extended-spectrum β-lactamase gene *bla*_TEM-1_ (41%).

**CONCLUSIONS:**

An unexpectedly high incidence of CRAB infections occurred in Polish hospitals. There is a need for effective CRAB prevention and control that includes effective hospital screening, national surveillance, and improved treatment options.

## INTRODUCTION

*Acinetobacter* spp. are nonfermenting gram-negative opportunistic pathogens that can colonize the skin, respiratory system, and gastrointestinal tract. They are responsible for many health care–associated infections, including ventilator-associated pneumonia and bloodstream infections (BSIs), especially in patients hospitalized in intensive care units (ICUs).^1^ Their ability to evade the host immune system significantly complicates efforts to manage and treat these infections.^2^ Since 2017, carbapenem—resistant *Acinetobacter baumannii* (CRAB) has consistently remained a critical priority pathogen and urgent threat to the public according to the World Health Organization and the United States (US) Centers for Disease Control and Prevention (CDC).^3,4^

Importantly, the epidemiology of *Acinetobacter* spp. can vary greatly by region. In the European Union / European Economic Area, approximately one-third of all *Acinetobacter* spp. isolates between 2017 and 2021 were carbapenem—resistant.^5^ After the peak of the COVID-19 pandemic in 2021, the number of reported *Acinetobacter* spp. cases resistant to carbapenems, fluoroquinolones, and aminoglycosides more than doubled, as compared with the 2018−2019 average. The greatest increases in *Acinetobacter* spp. antimicrobial resistant (AMR) cases were reported by countries that already had high AMR prevalence, such as Poland.^5^

In the US, incidence of CRAB has decreased from 3.3 per 10 000 hospitalizations in 2012 to 2.47 in 2017.^4^ The decrease is generally attributed to improved infection control and antibiotic stewardship practices that have become requirements for hospital accreditation in the US.^6^ There is concern this progress has been hampered by the COVID-19 pandemic, which led to increased antibiotic use and breakdown of infection control practices leading to reports of CRAB outbreaks.^7^ The global impact of CRAB is in part due to the ability of its resistance genes to spread via mobile genetic elements, as well as widespread antibiotic use.^2^ This has a negative impact on patient outcomes, as the mortality rate of *A. baumannii* infections ranges from 45% to 70%. One of its main predictors is carbapenem resistance,^1^ and carbapenems are frequently an empiric treatment choice for infections in areas such as Poland, where the prevalence of extensively drug-resistant organisms was 22.6% in the ICUs and 14.8% in non-ICUs,^8^ leading to delays in optimal antibiotic coverage for CRAB infections. This is further emphasized by CRAB being the fifth leading cause of death from resistant pathogens worldwide.^9^

In terms of resistance, *A. baumannii* employs a variety of mechanisms to reduce the effectiveness of antibiotic therapy. They include the use of efflux pumps, changes to antibiotic active sites, reduction of cell membrane permeability, and, probably the most common, the use of enzymes to inactivate antibiotics, particularly β-lactamases.^10^ *A*. *baumannii* has 2 intrinsic types of β-lactamases: AmpC-type cephalosporinases (no effect on extended-spectrum cephalosporin efficacy) and oxacillinases, mainly represented by the OXA-51-like variants^11,12^ and belonging to the OXAAb enzyme group, including OXA-66.^13^ In addition to the intrinsic β-lactamases, other β-lactamases have also been identified in *A. baumannii* as a source of resistance to carbapenems, including OXA-23, OXA-40, OXA-48, and OXA-58.^14^ The mechanisms of resistance related to carbapenemase production can be classified into 3 groups: Ambler class A (plasmid-mediated *KPC* and chromosomal *IMI*, *SME*, *GES*, and *NMC-A* genes), Ambler class B (metallo-β-lactamases whose genes are located on integrons and plasmids [*NDM*, *IMP*, *VIM*, *GIM*, *SPM*, and *SIM* genes]), and Ambler class D (plasmid-mediated oxacillinases, eg, OXA-48 or OXA-23).^15^ Prevalence of the resistance genes is geographically-specific, which results in difficulty generalizing local studies to other regions, and highlights the importance of local and regional studies to guide locally-relevant optimal treatment approaches.

Treatment of CRAB is difficult both in terms of diagnosis and antibiotic therapy. Rapid diagnostics (eg, based on nucleic acid amplification tests) to confirm infections with CRAB are often lacking in areas with high CRAB prevalence, which may result in delayed administration of appropriate antibiotics and negatively affect patient outcomes.^16^ The most common CRAB-related infection is pneumonia,^17^ and there are specific pharmacokinetic / pharmacodynamic properties of antibiotics used against CRAB that result in difficulty achieving appropriate drug levels in lung tissues.^18,19^ In addition, minimum inhibitory concentration (MIC) breakpoints for antibiotics against CRAB differ by organization^20,21^ (the European Committee on Antimicrobial Susceptibility Testing [EUCAST] recommendations apply in Poland) or do not exist, posing challenges for antibiotic choice and dosing. In other infections, especially those involving medical devices, such as endotracheal or tracheostomy tubes, CRAB’s ability to form biofilms can prevent antibiotics from penetrating and reaching the pathogen.^22,23^

In this study, we evaluated the resistance profiles of CRAB in 4 hospitals in southern Poland to characterize the prevalence of various CRAB β-lactamases and to inform regional empiric therapies for these difficult-to-treat infections. We also commented on the utility of commercial rapid tests in CRAB surveillance upon hospital admission. The resistance genes identified by these tests are not commonly detected in CRAB. Consequently, there is a potential risk of missing CRAB infections or colonization when these tests are utilized.

## PATIENTS AND METHODS

This laboratory-based study was carried out at the University Hospital in Kraków, St. Luke’s Provincial Hospital in Tarnów, the Provincial Hospital No. 5 in Sosnowiec, and the District Hospital in Bochnia.

Ethical approval was waived by the Bioethics Committee of the Jagiellonian University in view of the retrospective nature of the study, all procedures being performed as part of routine care, and the analysis not including any participant—identifying data. All data analyzed during this study were anonymized prior to the analysis. As a result, no informed consent was required from the participants.

The samples were collected between July and December 2022. A total number of admissions was 70 859. During the study, a multiplex polymerase chain reaction (PCR) (GeneXpert System, Cepheid, Sunnyvale, California, United States) was used for screening of the carbapenemase genes (types: *bla*_VIM_, *bla*_NDM_, *bla*_IMP_, *bla*_KPC_, *bla*_OXA-48_) to confirm the presence of carbapenemase—producing Enterobacterale (CPE) isolates on admission to the hospital in the cases of suspected CPE colonization (eg, antibiotic therapy, previous hospitalization, stay in long-term care facilities). A bacterial health care–associated infection (HAI) in adult patients was defined as a symptomatic infection diagnosed (or recognized) over 48 hours since the hospital admission. HAI cases were analyzed retrospectively using definitions from the Healthcare-Associated Infections Surveillance Network,^24^ which included BSIs, pneumonia, and urinary tract infections. As recommended by the European Centre for Disease Prevention and Control, bacterial diagnostic testing and its interpretation were performed according to the specimen type.

Some of the specimens were analyzed quantitatively; these included urine and positive quantitative cultures from minimally or possibly contaminated lower respiratory tract (LRT) specimens. Interpretation of quantitative results in mono- and polymicrobial cultures depended on the relative quantity of each microorganism. For a urine culture to be positive, the sample was required to have no more than 2 organism species and at least 10^5^ colony forming units (CFUs) per milliliter. For an LRT culture to be positive, bronchoalveolar lavage (BAL) was required to have at least 10^4^ CFU/ml or at least 5% of BAL—obtained cells needed to contain intracellular bacteria on direct microscopic examination, specimens from the LRT collected with a Wimberley protected brush were required to have at least 10^3^ CFU/ml, distal protected aspirate needed at least 10^3^ CFU/ml, and endotracheal aspirate needed at least 10^6^ CFU/ml.^24^

Microbiologic samples were obtained from the sites of infection. Only laboratory-confirmed *A. baumannii* HAI cases based on culture growth were qualified for the analysis; only the first isolate from each HAI case was analyzed. During the study period, 120 HAI cases (eg, pneumonia) were identified as caused by *A. baumannii*. As many as 107 out of the 120 isolates (89.2%) were identified as CRAB, and 82 CRAB isolates were analyzed (25 isolates were lost to follow—up for reasons independent from the authors).

The isolated organisms were identified by matrix-assisted laser desorption / ionization time-of-flight mass spectrometry (Bruker, Billerica, Massachusetts, United States or VITEK MS, bioMérieux, Craponne, France). Antibiotic susceptibility tests were performed by the hospital diagnostic laboratories using the automatic VITEK 2 method (bioMérieux), Phoenix M50 system (Becton Dickinson, Sparks, Maryland, United States), disc diffusion (Oxoid, Basingstoke, United Kingdom), MIC test strips (Liofilchem, Roseto degli Abruzzi, Italy), or broth microdilution in the case of colistin (MIC stripped plates, Diagnostics, Galanta, Slovakia), according to the EUCAST guidelines.^20^

The GeneMATRIX bacterial and yeast genomic DNA purification kit (EURx, Gdańsk, Poland) was used to extract genomic DNA from the CRAB isolates following the manufacturer’s protocol. The concentration and purity of the isolated DNA were assessed using a Nano Drop Lite spectro-photometer (Thermo Fisher Scientific, Waltham, Massachusetts, United States). DNA extracted from pure cultures was stored at −20 °C for further analyses.

The carbapenemase genes were identified using multiplex PCR (*bla*_VIM_, *bla*_OXA-48_, *bla*_OXA-23_, *bla*_KPC_, *bla*_NDM_, *bla*_OXA-40_, *bla*_OXA-58_, *bla*_IMP_, *bla*_GIM_, *bla*_GES_, *bla*_OXA-51_, *bla*_IMI_, and *bla*_VIM_)^25^ and real-time PCR (*bla*_OXA-66_ and *bla*_TEM_).^26^ PCR amplification was performed using the Color OptiTaq PCR Master Mix (EURx) in a final volume of 25 μl and a final primer concentration of 0.1 μl for each primer. Bacterial DNA served as a template. For the multiplex PCR, the reaction was conducted with an initial denaturation step of 3 minutes at 94 °C, followed by 30 cycles of 30 seconds at 94 °C, 15 seconds at 58 °C, and 1 minute at 72 °C for amplification. A final extension step of 5 minutes at 72 °C was performed. The PCR products were analyzed via gel electrophoresis. For the real-time PCR, cycling was carried out at 50 °C for 2 minutes, and then 95 °C for 2 minutes, followed by 40 cycles at 95 °C for 15 seconds, 55 °C for 15 seconds, and 72 °C for 1 minute (primer data are described in Supplementary material, Table S1). The datasets analyzed during the current study are available from the corresponding author upon reasonable request.

### Statistical analysis

In the statistical analysis, relative and absolute frequencies were used for nominal variables (described as number and percentage), and mean values with SD were used for quantitative variables. For data with non—normal distribution, median with interquartile range (IQR) was used. For independent samples with nominal variables, the χ^2^ test was employed. The Yates correction was used for 2 × 2 tables of nominal variables (when the expected values were <5) and the Fisher exact test was used for 2 × (n) tables (when the expected values were <5 and there was a small overall sample size [n <40]). For continuous data with normal distribution, the *t* test was used. For continuous variables without normal distribution and independent samples, the Mann–Whitney test was performed.

The analyses were carried out with the International Business Machines Corporation Statistical Package for the Social Sciences, version 29 (IBM Corporation, Armonk, New York, United States). In all analyses, the significance level was set at a *P* value below 0.05.

## RESULTS

In total, 82 CRAB isolates collected from the patients with *A. baumannii* infection were analyzed. Median (IQR) age of the patients was 65 (ICU, 65 [63–73]; non-ICU, 68 [52–80]) years (TABLE 1). Sources of infection included LRT (n = 38), bloodstream (n = 23), urinary tract (n = 13), and others (n = 8; including 6 wounds and 2 cerebrospinal fluid sources). Fifty-two CRAB isolates were collected from ICU patients and the most common CRAB sources in the ICU were LRT infections (n = 28; 54%) and BSIs (n = 14; 27%). In the non-ICU setting, 30 CRAB isolates were collected, and the most common CRAB sources were also LRT infections (n = 10; 33%) and BSIs (n = 9; 30%).

Depending on the hospital, the incidence rate of CRAB infections ranged from 428.6 to 759.5 per 10 000 admissions in ICUs, and from 0.3 to 21 per 10 000 admissions in non-ICUs. The proportion of CRAB isolates (in particular units) ranged from 81.8% to 100% in the ICUs and 75.7% to 100% in the non-ICUs (TABLE 1).

The most prevalent *bla*_OXA_ genes were *bla*_OXA-66–1_ (95%), *bla*_OXA-40_ (71%), *bla*_OXA-23_ (24%), and *bla*_OXA-51_ (12%). The *bla*_NDM_ genes were detected in 2% of the isolates, and *bla*_TEM-1_ genes were detected in 41% of the isolates. No *bla*_OXA-48_, *bla*_OXA-58_, *bla*_VIM_, *bla*_KPC_, *bla*_IMI_, *bla*_GES_, *bla*_GIM_, or *bla*_IMP_ genes were detected (TABLE 2). The *bla*_OXA-23_ gene was found more often in men than in women (32.2% vs 4.3%; *P* = 0.019).

Only 4 isolates had a single gene encoding carbapenemases, while the other had multiple β-lactamase genes. As many as 43 isolates had 2 genes, 29 had 3, and 5 had 4. Frequency of the detected gene patterns is shown in TABLE 3 (additional data are provided in Supplementary material, Table S2).

CRAB antibiotic susceptibility was the highest for cefiderocol (100%), colistin (96%), tigecycline (77%), gentamicin (51%), and ampicillin / sulbactam (36%). Less than 4% susceptibility was found in the case of ciprofloxacin, levofloxacin, meropenem, imipenem, trimethoprim / sulfamethoxazole, piperacillin / tazobactam, piperacillin, and imipenem / relebactam (TABLE 4) (additional data are provided in Supplementary material, Table S3). Statistical analysis did not reveal significant differences among the sources of infection (blood, urine, or LRT), and susceptibility of CRAB to individual antibiotics.

## DISCUSSION

The observed prevalence and incidence of CRAB in the studied hospitals significantly exceeded expected values. In addition, CRAB resistance was high against most antibiotics, and the antibiotics with better susceptibility profiles are generally associated with toxicities (eg, colistin). This highlights an urgent need to prevent CRAB infections, including avoiding the spread of CRAB, and the need for new treatments against CRAB infections.

Most *A. baumannii* isolates harbored multiple carbapenemase genes highlighting their ability to use many resistance mechanisms. The most common combination was *bla*_OXA-66–1_ with *bla*_OXA-40_. The *bla*_OXA-66–1_ gene (part of the *bla*_OXA-51-like_ gene family) is intrinsic to *A. baumannii*.^12^ At appropriate levels of expression, it has been associated with high levels of imipenem resistance in *A. baumannii*,^27^ but there is evidence that the degree of carbapenem resistance can be associated with the expression of other genes and resistance mechanisms within the pathogen.^28^ In our sample of carbapenem-resistant organisms, *bla*_OXA-66–1_ was found with another β-lactamase gene in all but 4 organisms, indicating possible reliance on other enzymes for carbapenem resistance. The *bla*_OXA-40_ gene prevalence has previously been described in southern Poland for the years 2005–2010, when 51 out of 104 isolates (49%) harbored this gene.^29^ Its enzymatic product is capable of hydrolyzing carbapenems as well as other β-lactams.^28^ As many as 58% of our isolates (54.2%) contained the *bla*_OXA-40_ genes, thus showing approximately a 5% point increase in its prevalence. This is a worrying phenomenon, which indicates that the prevalence of *bla*_OXA-40_ in Poland is approximately 3-fold higher than in the rest of Europe.^30^ Almost half of the studied isolates (41%), harbored *bla*_TEM_ genes. The *TEM* genes typically confer resistance to penicillins and early cephalosporins but have expanded their activity to include resistance against second-, third- and fourth-generation cephalosporins, monobactam, and β-lactamase inhibitors.^31^ The *TEM* genes are found at high frequencies in hospitals and clinics around the world,^32^ and often co-occur with other chromosomal (*AmpC*) or plasmid-mediated (*SHV*, *OXA*, *CTX-M*) β-lactamase encoding genes.^33^ The 24% prevalence of *bla*_OXA-23_ in our sample is 3-fold lower than in the rest of Europe, where *bla*_OXA-23_ genes were identified in 74.5% of analyzed *A. baumannii* isolates.^30^ Of note, commercially available rapid diagnostic tests only cover 5 enzymes (or their genes) (*VIM*, *NDM*, *IMP*, *KPC*, and *OXA-48*). Unfortunately, these genes / enzymes are not the major driver of *A. baumannii* resistance (as noted in our study), complicating efforts to detect the resistance mechanisms in *A. baumannii*.

*A. baumannii* is inherently difficult to treat, as it exhibits a variety of resistance mechanisms. In CRAB, the resistance to carbapenems is often associated with resistance to other categories of antibiotics, such as fluoroquinolones.^34^ Thus, the aim of this study was to provide clinicians with insight on empiric antibiotic regimens in Poland. There is no clear answer which antibiotic or which combination of antibiotics is a superior choice in treatment of CRAB infections. Yet, even with this uncertainty, the guidelines of both the European Society of Clinical Microbiology and Infectious Diseases (ESCMID) and the Infectious Diseases Society of America (IDSA) are mostly consistent in their CRAB treatment recommendations; they recommend the use of a combination therapy for treating CRAB infections.^35,36^ This increases the likelihood that one of the agents is effective against CRAB, given the lack of efficacy of single antibiotic regimens. Many CRAB infections occur in the ICU, where severely ill patients are at high risk for these infections and increased antibiotic use predisposes them to infections with resistant pathogens.^37^ The ICU setting often involves advanced life support interventions, such as continuous renal replacement therapy and extracorporeal membrane oxygenation, further complicating antibiotic treatment and dosing considerations due to diffusion, convection, and ultrafiltration mechanisms that alter antimicrobial concentrations in tissues.^38^

The IDSA guidelines recommend the use of sulbactam as the basis in most treatment regimens,^35^ regardless of the susceptibility profile of ampicillin / sulbactam.^39^ Only 36% of our CRAB isolates were susceptible to ampicillin / sulbactam, so in accordance with the IDSA guidelines, most isolates would need to be treated with high-dose ampicillin / sulbactam (9 g of sulbactam component) over an extended infusion time due to ampicillin / sulbactam resistance.^35^

ESCMID, contrary to IDSA, recommends ampicillin / sulbactam only when susceptibility is confirmed. Polymyxin (colistin) or high-dose tigecycline, if an isolate is susceptible in vitro, can be used when resistance to ampicillin / sulbactam occurs.^36^ Our isolates exhibited relatively high susceptibility to these antibiotics (colistin 96% and tigecycline 77%). Unfortunately, their use is not free from issues. Colistin-related toxicities include kidney injury and poor lung penetration,^16,40^ and the most recent Clinical and Laboratory Standards Institute update on colistin breakpoints does not include reference MIC values,^21^ though EUCAST does.^20^ The use of tigecycline against CRAB infections needs to be considered in the context of limited clinical data (ESCMID suggests its use in complicated intra-abdominal infections and in skin and soft tissue infections)^36^. Cefiderocol is an option against CRAB, especially for pneumonia,^41^ and has been suggested in combination with sulbactam.^16^ Our isolates were equally susceptible to cefiderocol but there were issues with laboratory techniques and obtaining accurate cefiderocol MICs.^42^ Studies have not found an association between the *bla*_OXA_ genes and cefiderocol resistance,^43^ which is reflected in 100% sensitivity of our isolates to cefiderocol. Fluoroquinolones are not recommended by IDSA, and would not be useful given low susceptibility of our isolates.^35^ The ESCMID guidelines do not refer to fluoroquinolones in CRAB treatment.^36^ Thus, the combination antibiotic regimen of choice depends on multiple factors that include the site of infection, pharmacokinetic / pharmacodynamic considerations, and the pathogen’s susceptibility profile. For example, Shields et al^16^ recommend ampicillin / sulbactam with tigecycline or cefiderocol for pneumonia and ampicillin / sulbactam with polymyxin B or cefiderocol for BSIs.

The use of meropenem in combination with other antibiotics has fallen out of favor. There were 2 clinical trials comparing colistin and colistin plus meropenem,^44,45^ which showed no difference between the colistin arm and the combination of meropenem with colistin in patients with CRAB. This suggests the use of meropenem did not add to the efficacy of colistin even when dosed appropriately and over an extended infusion period. Thus, the IDSA guidelines do not recommend the use of carbapenems in CRAB infections.^35^ ESCMID allows for administration of high-dose extended-infusion of carbapenem when the MIC is equal to or below 8 μg/l but as part of a combination therapy. It is worth noting that this is a good practice statement and expert opinion.^36^

New treatments against CRAB are thus urgently needed. Some of the most recently introduced agents are sulbactam / durlobactam. They were studied, in combination with imipenem / cilastatin, in a clinical noninferiority trial^46^ that showed higher mortality outcomes for colistin with imipenem / cilastatin than sulbactam / durlobactam with imipenem / cilastatin. The active component in sulbactam / durlobactam combination is sulbactam; durlobactam was added to inactivate OXA carbapenemases, which allows sulbactam to reach its target. The sulbactam / durlobactam combination has been recently approved by the US Food and Drug Administration against *A. baumannii* pneumonia but further data are needed to determine whether it should be used as monotherapy or in combination with other active antibiotics.^47^ Due to novelty of this therapy, we were unable to include sulbactam / durlobactam in our study, and this combination has not yet been approved for clinical use in Europe. The CDC indicates that other β-lactam / β-lactam inhibitors, meropenem / vaborbactam, can be effective in resistant gram-negative infections, but specifically in infections caused by CPE that express KPC and OXA-48–like enzymes, not in CRAB infections.^48^

Given difficult treatment of CRAB infections, prevention is a key component in their surveillance. Screening for CRAB can decrease prevalence of CRAB infections in an ICU by reducing transmission between patients when early isolation is implemented.^49^ It is important to consider which body site to utilize for CRAB screening, since the sites harbor isolates of different sensitivity.^50^ The skin swab isolates show the highest sensitivity (92% sensitivity from skin alone and 99% sensitivity from a combination of buccal mucosal and skin samples). Isolates collected from other sites can show a high rate of false-negative results (eg, 50% sensitivity in detecting CRAB colonization from the rectal site).^50^ The prevalence of CRAB colonization in our hospitals is unknown since screening is not routinely performed.

Carbapenem resistance markers found on routine early detection tests are common in Enterobacterales but not in *A. baumannii*, limiting utility of these methods in CRAB detection. These early-detection tests include phenotype-based methods detecting the activity of selected carbapenemases (eg, rapid colorimetric methods), immunochromatographic tests, and molecular methods (mainly based on polymerase chain reaction). The main limitation preventing their use is the small number of detected carbapenemases (only KPC, NDM, OXA-48, IMP, and VIM, or the genes encoding them). All of them are characteristic for the Enterobacterales order^51^; almost none of them occurred in the *A. baumannii* isolates we tested. Only 5% of the tested isolates (4 of 82) had at least 1 gene encoding these carbapenemases. Other tests, detecting OXA-23, OXA-40, and OXA-58 dedicated to *A. baumannii*, can be used for screening, but this still does not fully solve the surveillance problem of CRAB. Hence, infection control and prevention of carbapenemase—producing microorganisms, including CRAB infections is limited to nonspecific methods, such as hand hygiene.

### Limitations

Poland does not have an effective national system of surveillance for AMR,^52^ which limits comparability of the current data with the rest of the country and restricts the possibility of properly assessing temporal trends. Such systems are crucial to understand the epidemiology of CRAB and to evaluate the effect of interventions to slow its spread. Our samples came from 4 hospitals of different type but from the same geographic area, which limits generalizability of this study to other parts of Poland. These limitations provide opportunities for further research into the epidemiology of CRAB in Poland and support the need for a national plan to better understand and curb antimicrobial resistance.

## Conclusions

CRAB is an urgent public health threat. It is widespread throughout the world and is a major pathogen in Polish hospitals. Understanding its epidemiology, resistance mechanisms, and antibiotic susceptibility is crucial to implement focused infection control and antibiotic stewardship techniques and to help inform empiric and directed antibiotic treatments. This study provides up-to-date data on CRAB epidemiology in southern Poland, and interprets international treatment guidelines in the regional context. It indicates that southern Poland’s CRAB prevalence is higher, as compared with the rest of Europe, and is probably rising. This is concerning, since the antibiotic susceptibility profiles established in this study showed that the isolates are highly resistant to most used antibiotics, regardless of the infection site, leaving patients with less effective and more toxic treatment options. The genes involved in CRAB resistance are also not detected by routine surveillance tests, further complicating surveillance efforts. Thus, our study highlights the need for effective AMR prevention initiatives, better diagnostic surveillance tests, and improved treatment options against this difficult-to-treat pathogen.

## Supplementary Material

CRAB Poland Supplement

## Figures and Tables

**TABLE 1:** Patient demographics and epidemiology of *Acinetobacter baumannii* hospital-acquired infections in southern Poland between June and December 2022

Parameter	ICU (n = 52)	non-ICU (n = 30)	*P* value
Demographic data			
Age, y, median (IQR)	65 (63–73)	68 (52–80)	0.44
Sex, n (%)			
Male	42 (80.8)	17 (56.7)	0.04
Female	10 (19.2)	13 (43.3)	
Admissions, n (%)			
Tarnów	199 (21.4)	14 114 (20.1)	<0.001
Sosnowiec	210 (22.6)	13 323 (19.1)	
Kraków	441 (47.5)	31 398 (44.9)	
Bochnia	79 (8.5)	11 095 (15.9)	
Non-CRAB infections, n (%)			
Tarnów	1 (25)	0 (0)	0.15
Sosnowiec	2 (50)	9 (100)	
Kraków	0 (0)	0 (0)	
Bochnia	1 (25)	0 (0)	
CRAB infections^a^, n (%)			
Tarnów	12 (21.4)	20 (39.2)	<0.001
Sosnowiec	9 (16.1)	28 (54.9)	
Kraków	29 (51.8)	1 (2)	
Bochnia	6 (10.7)	2 (4)	
Prevalence of CRAB, %			
Tarnów	92.3	100	0.73
Sosnowiec	81.8	75.7	
Kraków	100	100	
Bochnia	85.7	100	
Incidence rate of CRAB infections (10 000 admissions)			
Tarnów	603	14.2	<0.001
Sosnowiec	428.6	21	
Kraków	657.6	0.3	
Bochnia	759.5	1.8	

a CRAB infections by hospital type are provided for 82 out of the 107 isolates identified as CRAB.

Abbreviations: CRAB, carbapenem-resistant *Acinetobacter baumannii*; ICU, intensive care unit; IQR, interquartile range

**TABLE 2:** β-Lactamase gene prevalence in 82 carbapenem-resistant *Acinetobacter baumannii* isolates from 4 hospitals in southern Poland between June and December 20*22*

Gene^a^	ICU (n = 52), n (%)	Non-ICU (n = 30), n (%)	*P* value
*bla*_OXA-23_	15 (28.8)	5 (16.7)	0.33
*bla*_OXA-40_	33 (63.5)	25 (83.3)	0.1
*bla*_OXA-51_	10 (19.2)	0	0.03
*bla*_OXA-66-1_	52 (100)	26 (86.7)	0.03
*bla*_NDM_	2 (3.8)	0	0.73
*bla*_TEM_	17 (32.7)	17 (56.7)	0.06

a There were no carbapenem-resistant *Acinetobacter baumannii* isolates with the *bla*_OXA-48_, *bla*_OXA-58_, *bla*_GES_, *bla*_GIM_, *bla*_IMI_, *bla*_IMP_, *bla*_KPC_, and *bla*_VIM_ genes.

Abbreviations: see TABLE 1

**TABLE 3:** Prevalence of detected gene patterns in 82 carbapenemase-producing *Acinetobacter baumannii* isolates from health care–associated infections at 4 hospitals in southern Poland between June and December 2022

Gene pattern	Number of strains	%
*bla*_OXA-40_, *bla*_OXA-66-1_	27	32.9
*bla*_OXA-40_, *bla*_OXA-66-1_, *bla*_TEM_	22	26.8
*bla*_OXA-23_, *bla*_OXA-66-1_	8	9.8
*bla*_OXA-23_, *bla*_TEM_	4	4.9
*bla*_OXA-66-1_	4	4.9
*bla*_OXA-40_, *bla*_OXA-66-1_, *bla*_OXA-51_	2	2.4
*bla*_OXA-40_, *bla*_OXA-23_, *bla*_OXA-66-1_	2	2.4
*bla*_OXA-23_, *bla*_OXA-66-1_, *bla*_OXA-51_	2	2.4
*bla*_OXA-40_, *bla*_OXA-66-1_, *bla*_OXA-51_, *bla*_TEM_	2	2.4
*bla*_OXA-66-1_, *bla*_TEM_	2	2.4
*bla*_OXA-40_, *bla*_OXA-23_, *bla*_OXA-66-1_, *bla*_TEM_	2	2.4
*bla*_OXA-40_, *bla*_OXA-23_, *bla*_OXA-66-1_, *bla*_OXA-51_	1	1.2
*bla*_OXA-23_, *bla*_OXA-66-1_, *bla*_OXA-51_, *bla*_NDM_, *bla*_TEM_	1	1.2
*bla*_OXA-66-1_, *bla*_OXA-51_	1	1.2
*bla*_OXA-66-1_, *bla*_OXA-51_, *bla*_TEM_	1	1.2
*bla*_OXA-66-1_, *bla*_NDM_	1	1.2

**TABLE 4:** Antibacterial susceptibility^a^ of 82 carbapenemase-producing *Acinetobacter baumannii* isolates collected from patients with health care–associated infections at 4 hospitals in southern Poland between June and December 2022 by the sampling source

Antibiotics	Susceptibility, %			
	Total (n = 82)^b^	Respiratory tract (n = 38)	Bloodstream (n = 23)	Urine (n = 13)
β-Lactams, penicillins				
Ampicillin / sulbactam	52.4	52.6	65.2	30.8
Piperacillin	0	0	0	0
Piperacillin / tazobactam	0	0	0	0
Carbapenems				
Imipenem	1.2	0	0	0
Imipenem / relebactam	0	0	0	0
Meropenem	2.4	0	4.3	0
Aminoglycosides				
Amikacin	24.4	26.3	21.7	23.1
Gentamicin	51.2	60.5	39.1	38.5
Tobramycin	19.5	23.7	13	15.4
Fluoroquinolones				
Ciprofloxacin	4.9	5.3	0	7.7
Levofloxacin	3.7	2.6	4.3	7.7
Other antibiotics				
Cefiderocol	100	100	100	100
Colistin	96.3	97.4	91.3	100
Minocycline	39	47.4	30.4	30.8
Tigecycline	76.8	78.9	73.9	69.2
Trimethoprim / sulfamethoxazole	2.4	2.6	4.3	0

a Antibiotic susceptibility results were interpreted using the European Committee on Antimicrobial Susceptibility Testing guidelines.^20^

b Includes 8 other sources

## WHAT’S NEW?

The genes encoding carbapenemases in carbapenem-resistant *Acinetobacter baumannii* (CRAB) vary not only globally but also regionally. These mechanisms of resistance are encountered frequently in southern Poland, but formal assessments of CRAB β-lactamases are lacking. Such knowledge has important implications for patient outcomes, as knowing the most prevalent CRAB resistance mechanisms in a region can guide initial therapy to ensure patients are started on optimal treatment as quickly as possible. The CRAB isolates in this study produced carbapenemases that are not identified on commercial rapid screening tests, so the usefulness of these tests in identifying CRAB for prevention and control is limited. Therefore, the use of such screening methods, specifically for carbapenem-producing organisms, can be misleading for clinical practitioners, and may lead to suboptimal empiric therapy choices.

## References

[1] Bartal C, Rolston KVI, Nesher L. Carbapenem-resistant *Acinetobacter baumannii*: colonization, infection and current treatment options. Infect Dis Ther. 2022; 11: 683–694. 10.1007/s40121-022-00597-w 35175509 PMC8960525

[2] Wong D, Nielsen TB, Bonomo RA, Clinical and pathophysiological overview of *Acinetobacter* infections: a century of challenges. Clin Microbiol Rev. 2017; 30: 409–447. 10.1128/CMR.00058-16 27974412 PMC5217799

[3] World Health Organization. WHO publishes list of bacteria for which new antibiotics are urgently needed. 2017. https://www.who.int/news/item/27-02-2017-who-publishes-list-of-bacteria-for-which-new-antibiotics-are-urgently-needed. Accessed December 10, 2023.

[4] Centers for Disease Control and Prevention (U.S.), National Center for Emerging Zoonotic and Infectious Diseases (U.S.). Division of Healthcare Quality Promotion. Antibiotic Resistance Coordination and Strategy Unit. Antibiotic resistance threats in the United States, 2019. https://stacks.cdc.gov/view/cdc/82532. Accessed December 10, 2023.

[5] European Centre for Disease Prevention and Control. Antimicrobial resistance in the EU/EEA (EARS-Net) - Annual Epidemiological Report 2021. Stockholm: ECDC. 2022. https://www.ecdc.europa.eu/sites/default/files/documents/AER-EARS-Net-2021_2022-final.pdf. Accessed December 15, 2023.

[6] Baker DW, Hyun D, Neuhauser MM, Leading practices in antimicrobial stewardship: conference summary. Jt Comm J Qual Patient Saf. 2019; 45: 517–523. 10.1016/j.jcjq.2019.04.006 31122789 PMC9552040

[7] Perez S, Innes GK, Walters MS, Increase in hospital-acquired carbapenem-resistant *Acinetobacter baumannii* infection and colonization in an acute care hospital during a surge in COVID-19 admissions - New Jersey, February-July 2020. MMWR Morb Mortal Wkly Rep. 2020; 69: 1827–1831. 10.15585/mmwr.mm6948e1 33270611 PMC7714028

[8] Pałka A, Kujawska A, Hareza DA, Secondary bacterial infections & extensively drug-resistant bacteria among COVID-19 hospitalized patients at the University Hospital in Kraków. Ann Clin Microbiol Antimicrob. 2023; 22: 77. 37620874 10.1186/s12941-023-00625-8 PMC10463524

[9] Antimicrobial Resistance Collaborators. Global burden of bacterial antimicrobial resistance in 2019: a systematic analysis. Lancet. 2022; 399: 629–655. 35065702 10.1016/S0140-6736(21)02724-0 PMC8841637

[10] Kyriakidis I, Vasileiou E, Pana ZD, Tragiannidis A. *Acinetobacter baumannii* antibiotic resistance mechanisms. Pathogens. 2021; 10: 373. 10.3390/pathogens10030373 33808905 PMC8003822

[11] Poirel L, Nordmann P. Carbapenem resistance in *Acinetobacter baumannii*: mechanisms and epidemiology. Clin Microbiol Infect. 2006; 12: 826–836. 10.1111/j.1469-0691.2006.01456.x 16882287

[12] Turton JF, Woodford N, Glover J, Identification of *Acinetobacter baumannii* by detection of the bla_OXA-51-like_ carbapenemase gene intrinsic to this species. J Clin Microbiol. 2006; 44: 2974–2976. 10.1128/JCM.01021-06 16891520 PMC1594603

[13] Takebayashi Y, Henderson SR, Chirgadze DY, OXA-66 structure and oligomerisation of OXAAb enzymes. Access Microbiol. 2022; 4: acmi000412. 10.1099/acmi.0.000412 PMC9675178 36415731

[14] Castanheira M, Mendes RE, Gales AC. Global epidemiology and mechanisms of resistance of *Acinetobacter baumannii-calcoaceticus* complex. Clin Infect Dis. 2023; 76: S166–S178. 10.1093/cid/ciad109 37125466 PMC10150277

[15] Logan LK, Weinstein RA. The epidemiology of carbapenem-resistant enterobacteriaceae: the impact and evolution of a global menace. J Infect Dis. 2017; 215: S28–S36. 10.1093/infdis/jiw282 28375512 PMC5853342

[16] Shields RK, Paterson DL, Tamma PD. Navigating available treatment options for carbapenem-resistant *Acinetobacter baumannii-calcoaceticus* complex infections. Clin Infect Dis. 2023; 76: S179–S193. 10.1093/cid/ciad094 37125467 PMC10150276

[17] Centers for Disease Control and Prevention. Emerging Infections Program, Healthcare-Associated Infections - Community Interface Surveillance Report, Multi-site Gram-negative Surveillance Initiative (MuGSI), Carbapenem-resistant Acinetobacter baumannii complex surveillance, 2021. https://www.cdc.gov/healthcare-associated-infections/media/pdfs/2021-CRAB-Report-508.pdf. Accessed April 6, 2024.

[18] Finazzi S, Luci G, Olivieri C, Tissue penetration of antimicrobials in intensive care unit patients: a systematic review—part I. Antibiotics. 2022; 11: 1164. 10.3390/antibiotics11091164 36139944 PMC9495190

[19] Viaggi B, Cangialosi A, Langer M, Tissue penetration of antimicrobials in intensive care unit patients: a systematic review—part II. Antibiotics. 2022; 11: 1193. 10.3390/antibiotics11091193 36139972 PMC9495066

[20] EUCAST. Clinical breakpoints - breakpoints and guidance. 2023. https://www.eucast.org/clinical_breakpoints. Accessed December 15, 2023.

[21] CLSI. CLSI M100-ED33:2023 Performance Standards for Antimicrobial Susceptibility Testing, 33rd Edition. 2023. https://clsi.org/media/tc4b1paf/m10033_samplepages-1.pdf. Accessed April 6, 2024.

[22] Upmanyu K, Haq QMohdR, Singh R. Factors mediating *Acinetobacter baumannii* biofilm formation: opportunities for developing therapeutics. Curr Res Microb Sci. 2022; 3: 100131. 10.1016/j.crmicr.2022.100131 35909621 PMC9325880

[23] Smitran A, Lukovic B, Bozic Lj, Carbapenem-resistant *Acinetobacter baumannii*: biofilm-associated genes, biofilm-eradication potential of disinfectants, and biofilm-inhibitory effects of selenium nanoparticles. Microorganisms. 2023; 11: 171. 10.3390/microorganisms11010171 36677463 PMC9865289

[24] European Centre for Disease Prevention and Control. Surveillance of healthcare-associated infections and prevention indicators in European intensive care units. Stockholm: ECDC. 2017. https://www.ecdc.europa.eu/en/publications-data/surveillance-healthcare-associated-infections-and-prevention-indicators-european. Accessed December 15, 2024.

[25] Cerezales M, Biniossek L, Gerson S, Novel multiplex PCRs for detection of the most prevalent carbapenemase genes in Gram-negative bacteria within Germany. J Med Microbiol. 2021; 70: 001310. 10.1099/jmm.0.001310 33448924

[26] Han L, Lei J, Xu J, Han S. blaOXA-23-like and blaTEM rather than blaOXA-51-like contributed to a high level of carbapenem resistance in *Acinetobacter baumannii* strains from a teaching hospital in Xi’an, China. Medicine. 2017; 96: e8965. 10.1097/MD.0000000000008965 29310399 PMC5728800

[27] Hu WS, Yao SM, Fung CP, An OXA-66/OXA-51-like carbapenemase and possibly an efflux pump are associated with resistance to imipenem in *Acinetobacter baumannii*. Antimicrob Agents Chemother. 2007; 51: 3844–3852. 10.1128/AAC.01512-06 17724156 PMC2151406

[28] Ramirez MS, Bonomo RA, Tolmasky ME. Carbapenemases: transforming *Acinetobacter baumannii* into a yet more dangerous menace. Biomolecules. 2020; 10: 720. 10.3390/biom10050720 32384624 PMC7277208

[29] Nowak P, Paluchowska P, Budak A. Distribution of blaOXA genes among carbapenem-resistant *Acinetobacter baumannii* nosocomial strains in Poland. New Microbiol. 2012; 35: 317–325. 22842601

[30] Mendes RE, Doyle TB, Kantro V, Cefiderocol in vitro activity against molecularly characterized Acinetobacter baumannii-calcoaceticus complex and Pseudomonas aeruginosa clinical isolates causing infection in Europe and adjacent regions (2020). 2022. https://www.jmilabs.com/data/posters/21-SHI-06-P2-CFDC-Mol-NF-ECCMID-2022V2.pdf?x17136. Accessed December 15, 2023.

[31] Bradford PA. Extended-spectrum beta-lactamases in the 21st century: characterization, epidemiology, and detection of this important resistance threat. Clin Microbiol Rev. 2001; 14: 933–951. 10.1128/CMR.14.4.933-951.2001 11585791 PMC89009

[32] Salverda MLM, De Visser JAGM, Barlow M. Natural evolution of TEM-1 β-lactamase: experimental reconstruction and clinical relevance. FEMS Microbiol Rev. 2010; 34: 1015–1036. 10.1111/j.1574-6976.2010.00222.x 20412308

[33] Park YJ, Park SY, Oh EJ, Occurrence of extended-spectrum beta—lactamases among chromosomal AmpC-producing *Enterobacter cloacae*, *Citrobacter freundii*, and *Serratia marcescens* in Korea and investigation of screening criteria. Diagn Microbiol Infect Dis. 2005; 51: 265–269. 10.1016/j.diagmicrobio.2004.11.009 15808318

[34] Tawfick MM, Adulall AK, El-Kholy AA, Manakhly ARE. Mutation-based fluoroquinolone resistance in carbapenem-resistant Acinetobacter baumannii and Escherichia coli isolates causing catheter-related bloodstream infections. Int J Health Sci (Qassim). 2023; 17: 18–25. PMC10155246 37151743

[35] Tamma PD, Aitken SL, Bonomo RA, Infectious Diseases Society of America 2023 Guidance on the treatment of antimicrobial resistant gram-negative infections. Clinical Infectious Diseases. 2023 Jul 18. [Epub ahead of print]. 10.1093/cid/ciad428 37463564

[36] Paul M, Carrara E, Retamar P, European Society of Clinical Microbiology and Infectious Diseases (ESCMID) guidelines for the treatment of infections caused by multidrug-resistant Gram-negative bacilli (endorsed by European Society of Intensive Care Medicine). Clin Microbiol Infect. 2022; 28: 521–547. 10.1016/j.cmi.2021.11.025 34923128

[37] Baditoiu L, Axente C, Lungeanu D, Intensive care antibiotic consumption and resistance patterns: a cross-correlation analysis. Ann Clin Microbiol Antimicrob. 2017; 16: 71. 10.1186/s12941-017-0251-8 29132352 PMC5683545

[38] Kühn D, Metz C, Seiler F, Antibiotic therapeutic drug monitoring in intensive care patients treated with different modalities of extracorporeal membrane oxygenation (ECMO) and renal replacement therapy: a prospective, observational single-center study. Crit Care. 2020; 24: 664. 10.1186/s13054-020-03397-1 33239110 PMC7689974

[39] Deng Y, Chen L, Yue M, Sulbactam combined with tigecycline improves outcomes in patients with severe multidrug-resistant *Acinetobacter baumannii* pneumonia. BMC Infect Dis. 2022; 22: 795. 10.1186/s12879-022-07778-5 36271362 PMC9585752

[40] Zhang J, Song C, Wu M, Physiologically-based pharmacokinetic modeling to inform dosing regimens and routes of administration of rifampicin and colistin combination against *Acinetobacter baumannii*. Eur J Pharm Sci. 2023; 185: 106443. 10.1016/j.ejps.2023.106443 37044198

[41] Shortridge D, Streit JM, Mendes R, Castanheira M. In vitro activity of cefiderocol against U.S. and European gram-negative clinical isolates collected in 2020 as part of the SENTRY Antimicrobial Surveillance Program. Microbiol Spectr. 2022; 10: e0271221. 10.1128/spectrum.02712-21 35262394 PMC9045385

[42] Hackel MA, Tsuji M, Yamano Y, In vitro activity of the siderophore cephalosporin, cefiderocol, against carbapenem-nonsusceptible and multidrug-resistant isolates of gram-negative bacilli collected worldwide in 2014 to 2016. Antimicrob Agents Chemother. 2018; 62: e01968–17. 10.1128/AAC.01968-17 29158270 PMC5786755

[43] Seifert H, Müller C, Stefanik D, In vitro activity of cefiderocol against a global collection of carbapenem-resistant *Acinetobacter baumannii* isolates. Antibiotics (Basel). 2023; 12: 1172. 10.3390/antibiotics12071172 37508268 PMC10376869

[44] Kaye KS, Marchaim D, Thamlikitkul V, Colistin monotherapy versus combination therapy for carbapenem-resistant organisms. NEJM Evid. 2023; 2: 10.1056/evidoa2200131. PMC10398788 37538951

[45] Paul M, Daikos GL, Durante-Mangoni E, Colistin alone versus colistin plus meropenem for treatment of severe infections caused by carbapenem-resistant Gram-negative bacteria: an open-label, randomised controlled trial. Lancet Infect Dis. 2018; 18: 391–400. 10.1016/S1473-3099(18)30099-9 29456043

[46] Kaye KS, Shorr AF, Wunderink RG, Efficacy and safety of sulbactam-durlobactam versus colistin for the treatment of patients with serious infections caused by *Acinetobacter baumannii-calcoaceticus* complex: a multicentre, randomised, active-controlled, phase 3, non-inferiority clinical trial (ATTACK). Lancet Infect Dis. 2023; 23: 1072–1084. 10.1016/S1473-3099(23)00184-6 37182534

[47] Food and Drug Administration (U.S.). FDA approves new treatment for pneumonia caused by certain difficult-to-treat bacteria. 2023. https://www.fda.gov/news-events/press-announcements/fda-approves-new-treatment-pneumonia-caused-certain-difficult-treat-bacteria. Accessed December 15, 2023.

[48] Ham DC, Mahon G, Bhaurla SK, Gram-negative bacteria harboring multiple carbapenemase genes, United States, 2012–2019. Emerg Infect Dis. 2021; 27: 2475–2479. 10.3201/eid2709.210456 34424168 PMC8386808

[49] Yamamoto N, Hamaguchi S, Akeda Y, Rapid screening and early precautions for carbapenem-resistant *Acinetobacter baumannii* carriers decreased nosocomial transmission in hospital settings: a quasi-experimental study. Antimicrob Resist Infect Control. 2019; 8: 110. 10.1186/s13756-019-0564-9 31297191 PMC6598269

[50] Nutman A, Temkin E, Lellouche J, Detecting carbapenem—resistant *Acinetobacter baumannii* (CRAB) carriage: which body site should be cultured? Infect Control Hosp Epidemiol. 2020; 41: 965–967. 10.1017/ice.2020.197 32618523 PMC7511923

[51] Simner PJ, Opene BNA, Chambers KK, carbapenemase detection among carbapenem-resistant glucose-nonfermenting Gram-negative bacilli. J Clin Microbiol. 2017; 55: 2858–2864. 10.1128/JCM.00775-17 28701421 PMC5648721

[52] Lötsch F, Albiger B, Monnet DL, Epidemiological situation, laboratory capacity and preparedness for carbapenem-resistant *Acinetobacter baumannii* in Europe, 2019. Euro Surveill. 2020; 25: 2001735. 10.2807/1560-7917.ES.2020.25.45.2001735 33183407 PMC7667627

