# Supplementary material for: Molecular epidemiology and clinical significance of carbapenemase genes in carbapenem-resistant Acinetobacter baumannii isolates in southern Poland
Source: Pol Arch Intern Med. Author manuscript; Available in PMC 2024 Dec 4. (PMC11615936; doi:10.20452/pamw.16734)
Supplement: CRAB Poland Supplement [file NIHMS2038342-supplement-CRAB_Poland_Supplement.pdf]

Supplementary material

Serwacki PA, Hareza DA, Kujawska A, et al. Molecular epidemiology and clinical significance of carbapenemase genes among carbapenem-resistant Acinetobacter baumannii *isolates in southern Poland. Pol Arch Intern Med. 2024; 134: 16734. doi:10.20452/pamw.16734*

Please note that the journal is not responsible for the scientific accuracy or functionality of any supplementary material submitted by the authors. An queries (except missing content) should be directed to the corresponding author of the article.

Table S1. Primers used to detect carbapenemase genes according to Cerezales M. et al. and Han L. et al. [25,26].

Detected Genes	Primer Sequences	Product Size (bp)	Annealing Temperature (°C)
bla$_{VIM}$	5′-GATGGTGTTTGGTCGCATATC-3′	202	58°C
bla$_{OXA-48}$	5′-GGTAGCAAAGGAATGGCAAGAA-3′	611	58°C
bla$_{OXA-23}$	5′-TCTGGTTGTACGGTTCAGCA-3′	718	58°C
bla$_{KPC}$	5′-CGCCAATTTGTTGCTGAAGG-3′	312	58°C
bla$_{NDM}$	5′-GTTTGATCGTCAGGGATGGC-3′	517	58°C
bla$_{OXA-40}$	5′-GCATTGTCAGCAGTTCCAGT-3′	402	58°C
bla$_{OXA-58}$	5′-ATCAAGAATTGGCACGTCGT-3′	303	58°C
bla$_{IMP}$	5′-GAAGGCGTTTATGTTCATAC-3′	587	58°C
bla$_{GIM}$	5′-TTATCCTGGGCGACTGACAG-3′	508	58°C
bla$_{GES}$	5′-CTCAGATCGGTGTTGCGATC-3′	416	58°C
bla$_{OXA-51}$	5′-TGTGGTAAGCACTTGATGGG-3′	303	58°C
bla$_{IMI}$	5′-AGACTCGATCGTTGGGAGTT-3′	206	58°C
bla$_{OXA-66}$	5’-TCGGCCTTGAGCACCATAAG-3’	n/a	55°C
bla$_{TEM}$	5’-ATAAAATTCTTGAAGACGAAA-3’	n/a	55°C

Abbreviations: bp, base pairs; n/a, not available in RT-PCR reaction

Table S2. Detected β-lactamase genes in 82 carbapenem-resistant *Acinetobacter baumannii* isolates from 4 hospitals in southern Poland from 06.2022–12.2022.

No	Source of infection[a]	*bla*$_{OXA-40}$	*bla*$_{OXA-23}$	*bla*$_{OXA-66-1}$	*bla*$_{OXA-51}$	*bla*$_{NDM}$	*bla*$_{TEM}$
1	LRT	*bla*$_{OXA-40}$		*bla*$_{OXA-66-1}$			
2	Urine	*bla*$_{OXA-40}$		*bla*$_{OXA-66-1}$			
3	LRT	*bla*$_{OXA-40}$		*bla*$_{OXA-66-1}$			
4	Blood	*bla*$_{OXA-40}$		*bla*$_{OXA-66-1}$			
5	LRT	*bla*$_{OXA-40}$		*bla*$_{OXA-66-1}$			
6	LRT	*bla*$_{OXA-40}$		*bla*$_{OXA-66-1}$			
7	LRT	*bla*$_{OXA-40}$		*bla*$_{OXA-66-1}$			
8	Urine	*bla*$_{OXA-40}$		*bla*$_{OXA-66-1}$			
9	Urine	*bla*$_{OXA-40}$		*bla*$_{OXA-66-1}$			

10	LRT	$bla_{\text{OXA-40}}$		$bla_{\text{OXA-66-1}}$			
11	LRT	$bla_{\text{OXA-40}}$		$bla_{\text{OXA-66-1}}$			
12	Urine	$bla_{\text{OXA-40}}$		$bla_{\text{OXA-66-1}}$			
13	LRT	$bla_{\text{OXA-40}}$		$bla_{\text{OXA-66-1}}$			
14	Blood	$bla_{\text{OXA-40}}$		$bla_{\text{OXA-66-1}}$			
15	LRT	$bla_{\text{OXA-40}}$		$bla_{\text{OXA-66-1}}$			
16	LRT	$bla_{\text{OXA-40}}$		$bla_{\text{OXA-66-1}}$			
17	Urine	$bla_{\text{OXA-40}}$		$bla_{\text{OXA-66-1}}$			
18	Blood	$bla_{\text{OXA-40}}$		$bla_{\text{OXA-66-1}}$			
19	LRT	$bla_{\text{OXA-40}}$		$bla_{\text{OXA-66-1}}$			
20	LRT	$bla_{\text{OXA-40}}$		$bla_{\text{OXA-66-1}}$			
21	LRT	$bla_{\text{OXA-40}}$		$bla_{\text{OXA-66-1}}$			
22	LRT			$bla_{\text{OXA-66-1}}$		bla_{NDM}	
23	LRT	$bla_{\text{OXA-40}}$		$bla_{\text{OXA-66-1}}$			
24	Urine	$bla_{\text{OXA-40}}$		$bla_{\text{OXA-66-1}}$			
25	Blood	$bla_{\text{OXA-40}}$		$bla_{\text{OXA-66-1}}$			
26	Blood	$bla_{\text{OXA-40}}$		$bla_{\text{OXA-66-1}}$			
27	LRT	$bla_{\text{OXA-40}}$		$bla_{\text{OXA-66-1}}$			
28	Other	$bla_{\text{OXA-40}}$		$bla_{\text{OXA-66-1}}$			bla_{TEM}
29	LRT	$bla_{\text{OXA-40}}$		$bla_{\text{OXA-66-1}}$			bla_{TEM}
30	Urine	$bla_{\text{OXA-40}}$		$bla_{\text{OXA-66-1}}$			bla_{TEM}
31	Other	$bla_{\text{OXA-40}}$		$bla_{\text{OXA-66-1}}$			bla_{TEM}
32	LRT	$bla_{\text{OXA-40}}$		$bla_{\text{OXA-66-1}}$			bla_{TEM}
33	LRT	$bla_{\text{OXA-40}}$		$bla_{\text{OXA-66-1}}$			bla_{TEM}
34	Other	$bla_{\text{OXA-40}}$		$bla_{\text{OXA-66-1}}$			bla_{TEM}
35	Urine	$bla_{\text{OXA-40}}$		$bla_{\text{OXA-66-1}}$			bla_{TEM}
36	LRT	$bla_{\text{OXA-40}}$		$bla_{\text{OXA-66-1}}$			bla_{TEM}
37	Blood	$bla_{\text{OXA-40}}$		$bla_{\text{OXA-66-1}}$			bla_{TEM}
38	LRT	$bla_{\text{OXA-40}}$		$bla_{\text{OXA-66-1}}$			bla_{TEM}
39	LRT	$bla_{\text{OXA-40}}$		$bla_{\text{OXA-66-1}}$			bla_{TEM}
40	Urine		$bla_{\text{OXA-23}}$				bla_{TEM}
41	Other		$bla_{\text{OXA-23}}$				bla_{TEM}
42	LRT		$bla_{\text{OXA-23}}$				bla_{TEM}
43	LRT		$bla_{\text{OXA-23}}$				bla_{TEM}
44	Urine	$bla_{\text{OXA-40}}$		$bla_{\text{OXA-66-1}}$			bla_{TEM}
45	Blood	$bla_{\text{OXA-40}}$		$bla_{\text{OXA-66-1}}$			bla_{TEM}
46	LRT	$bla_{\text{OXA-40}}$		$bla_{\text{OXA-66-1}}$			bla_{TEM}
47	LRT	$bla_{\text{OXA-40}}$		$bla_{\text{OXA-66-1}}$			bla_{TEM}
48	LRT	$bla_{\text{OXA-40}}$		$bla_{\text{OXA-66-1}}$			bla_{TEM}
49	LRT	$bla_{\text{OXA-40}}$		$bla_{\text{OXA-66-1}}$			bla_{TEM}
50	Blood	$bla_{\text{OXA-40}}$		$bla_{\text{OXA-66-1}}$			bla_{TEM}
51	LRT	$bla_{\text{OXA-40}}$		$bla_{\text{OXA-66-1}}$			bla_{TEM}

52	LRT	$bla_{\text{OXA-40}}$		$bla_{\text{OXA-66-1}}$			bla_{TEM}
53	Other	$bla_{\text{OXA-40}}$		$bla_{\text{OXA-66-1}}$			bla_{TEM}
54	Blood			$bla_{\text{OXA-66-1}}$			
55	Urine			$bla_{\text{OXA-66-1}}$			
56	Urine			$bla_{\text{OXA-66-1}}$			
57	Blood	$bla_{\text{OXA-40}}$		$bla_{\text{OXA-66-1}}$	$bla_{\text{OXA-51}}$		
58	Other		$bla_{\text{OXA-23}}$	$bla_{\text{OXA-66-1}}$			
59	LRT		$bla_{\text{OXA-23}}$	$bla_{\text{OXA-66-1}}$			
60	Blood	$bla_{\text{OXA-40}}$		$bla_{\text{OXA-66-1}}$			
61	Urine		$bla_{\text{OXA-23}}$	$bla_{\text{OXA-66-1}}$			
62	Blood		$bla_{\text{OXA-23}}$	$bla_{\text{OXA-66-1}}$	$bla_{\text{OXA-51}}$	bla_{NDM}	bla_{TEM}
63	LRT		$bla_{\text{OXA-23}}$	$bla_{\text{OXA-66-1}}$	$bla_{\text{OXA-51}}$		
64	LRT			$bla_{\text{OXA-66-1}}$			
65	LRT		$bla_{\text{OXA-23}}$	$bla_{\text{OXA-66-1}}$			
66	LRT		$bla_{\text{OXA-23}}$	$bla_{\text{OXA-66-1}}$			
67	Other		$bla_{\text{OXA-23}}$	$bla_{\text{OXA-66-1}}$			
68	Blood		$bla_{\text{OXA-23}}$	$bla_{\text{OXA-66-1}}$			
69	Blood	$bla_{\text{OXA-40}}$	$bla_{\text{OXA-23}}$	$bla_{\text{OXA-66-1}}$			
70	Other	$bla_{\text{OXA-40}}$		$bla_{\text{OXA-66-1}}$	$bla_{\text{OXA-51}}$		bla_{TEM}
71	Blood			$bla_{\text{OXA-66-1}}$			bla_{TEM}
72	LRT	$bla_{\text{OXA-40}}$	$bla_{\text{OXA-23}}$	$bla_{\text{OXA-66-1}}$	$bla_{\text{OXA-51}}$		
73	Blood			$bla_{\text{OXA-66-1}}$	$bla_{\text{OXA-51}}$		
74	Blood		$bla_{\text{OXA-23}}$	$bla_{\text{OXA-66-1}}$			
75	Blood		$bla_{\text{OXA-23}}$	$bla_{\text{OXA-66-1}}$	$bla_{\text{OXA-51}}$		
76	LRT	$bla_{\text{OXA-40}}$	$bla_{\text{OXA-23}}$	$bla_{\text{OXA-66-1}}$			
77	LRT	$bla_{\text{OXA-40}}$		$bla_{\text{OXA-66-1}}$	$bla_{\text{OXA-51}}$		
78	Blood			$bla_{\text{OXA-66-1}}$	$bla_{\text{OXA-51}}$		bla_{TEM}
79	Blood	$bla_{\text{OXA-40}}$	$bla_{\text{OXA-23}}$	$bla_{\text{OXA-66-1}}$			bla_{TEM}
80	Blood	$bla_{\text{OXA-40}}$		$bla_{\text{OXA-66-1}}$	$bla_{\text{OXA-51}}$		bla_{TEM}
81	Blood			$bla_{\text{OXA-66-1}}$			bla_{TEM}
82	Blood	$bla_{\text{OXA-40}}$	$bla_{\text{OXA-23}}$	$bla_{\text{OXA-66-1}}$			bla_{TEM}

[a]Other includes 6 wound and 2 cerebral spinal fluid sources

Abbreviations: LRT, lower respiratory tract

Table S3. Source of infection and antibiotic susceptibility testing[a] results in 82 carbapenem-resistant *Acinetobacter baumannii* isolates from 4 hospitals in southern Poland from 06.2022–12.2022.

No	Source of infection[b]	Ciprofloxacin	Levofloxacin	Gentamycin	Amikacin	Tobramycin	Imipenem	Meropenem	Ampicillin/sulbactam	Piperacillin/tazobactam	Piperacillin	Colistin	Trimetoprim/ sulphametoxazole	Tigecycline (MIC)	Tigecycline	Imipenem+Relebactam	Imipenem+Relebactam	Minocycline (MIC)	Minocycline	Cefiderocol (MIC)	Cefiderocol
1	LRT	R	R	R	R	R	R	R	R	R	R	S	R	1.5	S	32	R	8	I	0.19	S
2	Urine	R	R	R	R	R	R	R	I	R	R	S	R	3	R	32	R	16	R	0.5	S
3	LRT	R	R	R	R	R	R	R	R	R	R	S	R	3	R	32	R	24	R	0.25	S
4	Blood	R	R	R	S	R	R	R	R	R	R	S	R	0.75	S	32	R	12	I	0.09	S
5	LRT	R	R	S	R	S	R	R	R	R	R	S	R	1	S	32	R	8	I	0.5	S
6	LRT	R	R	S	S	S	R	R	S	R	R	S	R	0.75	S	32	R	8	I	0.38	S
7	LRT	R	R	R	S	R	R	R	R	R	R	S	R	0.5	S	32	R	12	I	0.09	S
8	Urine	R	R	R	S	R	R	R	R	R	R	S	R	0.75	S	32	R	8	I	0.06	S
9	Urine	R	R	R	R	R	R	R	R	R	R	S	R	1	S	32	R	16	R	0.06	S
10	LRT	R	R	S	S	S	R	R	R	R	R	S	R	1	S	32	R	16	R	0.38	S
11	LRT	R	R	R	R	R	R	R	R	R	R	S	R	1	S	32	R	16	R	0.09	S
12	Urine	R	R	R	R	R	R	R	R	R	R	S	R	2	R	32	R	16	R	1	S
13	LRT	R	R	R	R	R	R	R	R	R	R	S	R	4	R	32	R	16	R	0.5	S
14	Blood	R	R	R	R	R	R	R	R	R	R	S	R	1.5	S	32	R	24	R	0.13	S
15	LRT	R	R	R	R	R	R	R	R	R	R	S	R	1.5	S	32	R	16	R	0.13	S
16	LRT	R	R	R	R	R	R	R	R	R	R	S	R	1.5	S	32	R	24	R	0.5	S

17	Urine	R	R	R	R	R	R	R	R	R	R	S	R	1	S	32	R	12	I	0.5	S
18	Blood	R	R	R	R	R	R	R	R	R	R	S	R	2	R	32	R	16	R	0.5	S
19	LRT	S	S	S	S	S	R	R	S	R	R	S	R	0.75	S	32	R	4	S	0.25	S
20	LRT	R	R	R	R	S	R	R	R	R	R	S	R	1	S	32	R	16	R	0.19	S
21	LRT	R	R	R	R	R	R	R	I	R	R	S	R	3	R	32	R	16	R	3	S
22	LRT	R	R	R	S	R	R	R	R	R	R	S	R	1.5	S	32	R	12	I	0.25	S
23	LRT	R	R	R	S	R	R	R	S	R	R	S	R	0.38	S	32	R	4	S	0.5	S
24	Urine	R	R	R	R	R	R	R	R	R	R	S	R	1	S	32	R	16	R	0.13	S
25	Blood	R	R	R	R	R	R	R	R	R	R	S	R	1	S	32	R	12	I	0.94	S
26	Blood	R	R	S	S	S	R	R	S	R	R	S	R	1	S	32	R	16	R	0.75	S
27	LRT	R	R	S	S	S	R	R	S	R	R	S	R	0.75	S	32	R	12	I	0.25	S
28	Other	I	R	S	R	S	S	S	R	R	R	S	R	1	S	32	R	16	R	0.94	S
29	LRT	R	R	R	R	R	R	R	R	R	R	S	R	1	S	32	R	12	I	0.94	S
30	Urine	R	R	S	R	R	R	R	S	R	R	S	R	1.5	S	32	R	16	R	0.5	S
31	Other	R	R	R	R	R	R	R	I	R	R	S	R	3	R	32	R	4	S	0.25	S
32	LRT	R	R	R	R	R	R	R	I	R	R	S	R	1.5	S	32	R	12	I	0.25	S
33	LRT	R	R	S	R	R	R	R	I	R	R	S	R	0.75	S	32	R	3	S	0.5	S
34	Other	R	R	S	S	R	R	R	R	R	R	S	R	1.5	S	32	R	16	R	0.75	S
35	Urine	R	R	R	R	R	R	R	R	R	R	S	R	3	R	32	R	16	R	2	S
36	LRT	R	R	S	R	R	R	R	R	R	R	S	R	1	S	32	R	16	R	0.25	S

37	Blood	R	R	R	R	R	R	R	I	R	R	S	R	3	R	32	R	6	I	0.38	S
38	LRT	R	R	S	R	R	R	R	S	R	R	S	R	3	R	32	R	8	I	0.25	S
39	LRT	R	R	S	R	R	R	R	R	R	R	S	R	0.75	S	32	R	10	I	0.25	S
40	Urine	R	R	S	R	R	R	R	R	R	R	S	R	3	R	32	R	24	R	0.38	S
41	Other	R	R	S	R	R	R	R	R	R	R	S	R	1.5	S	32	R	0.24	S	1	S
42	LRT	R	R	S	R	R	R	R	I	R	R	S	S	4	R	32	R	8	I	0.25	S
43	LRT	R	R	S	R	R	R	R	I	R	R	S	R	0.5	S	32	R	3	S	0.38	S
44	Urine	R	R	R	R	R	R	R	I	R	R	S	R	0.75	S	32	R	12	I	1	S
45	Blood	R	R	S	R	R	R	R	I	R	R	S	R	3	R	32	R	16	R	0.25	S
46	LRT	R	R	S	R	R	R	R	R	R	R	S	R	1.5	S	32	R	24	R	0.38	S
47	LRT	R	R	S	R	R	R	R	S	R	R	S	R	25	R	32	R	12	I	0.5	S
48	LRT	R	R	R	R	R	R	R	R	R	R	S	R	2	R	32	R	32	R	0.38	S
49	LRT	R	R	S	R	R	R	R	I	R	R	R	R	2	R	32	R	16	R	0.25	S
50	Blood	R	R	R	R	R	R	R	R	R	R	S	R	1	S	32	R	24	R	0.5	S
51	LRT	R	R	S	R	R	R	R	R	R	R	S	R	1	S	32	R	16	R	0.75	S
52	LRT	R	R	S	R	R	R	R	S	R	R	S	R	0.5	S	32	R	1.5	S	0.75	S
53	Other	R	R	S	R	R	R	R	R	R	R	S	R	1	S	32	R	6	I	1	S
54	Blood	R	R	S	R	R	R	R	R	R	R	S	R	3	R	32	R	32	R	0.5	S
55	Urine	S	S	S	R	R	R	R	R	R	R	S	R	0.38	S	32	R	32	R	1.5	S
56	Urine	R	R	S	S	S	R	R	S	R	R	S	R	1	S	32	R	16	R	0.38	S

57	Blood	R	R	R	S	S	R	R	S	R	R	S	I	6	R	32	R	64	R	0.5	S
58	Other	R	R	S	S	S	R	R	S	R	R	S	R	1	S	32	R	16	R	0.38	S
59	LRT	R	R	S	R	R	R	R	S	R	R	S	R	1	S	32	R	24	R	2	S
60	Blood	R	R	S	S	S	R	R	R	R	R	S	R	1.5	S	32	R	24	R	1.5	S
61	Urine	R	R	S	S	S	R	R	R	R	R	S	R	1.5	S	32	R	8	I	0.13	S
62	Blood	R	R	S	S	R	R	R	S	R	R	S	R	1	S	32	R	16	R	0.19	S
63	LRT	R	R	S	R	R	R	R	I	R	R	S	R	1	S	32	R	24	R	0.75	S
64	LRT	R	R	S	S	S	R	R	S	R	R	S	R	0.75	S	32	R	24	R	0.38	S
65	LRT	S	R	S	S	S	R	R	R	R	R	S	R	1.5	S	32	R	32	R	1.5	S
66	LRT	R	R	S	S	S	R	R	S	R	R	S	R	1.5	S	32	R	24	R	0.25	S
67	Other	R	R	R	R	R	R	R	S	R	R	S	R	1	S	32	R	16	R	0.5	S
68	Blood	R	R	S	R	R	R	R	S	R	R	R	R	0.75	S	32	R	24	R	0.19	S
69	Blood	R	R	R	R	R	R	R	S	R	R	S	R	1.5	S	32	R	16	R	0.38	S
70	Other	R	R	R	R	R	R	R	S	R	R	S	R	1	S	32	R	16	R	0.25	S
71	Blood	R	R	R	R	R	R	R	S	R	R	S	R	1.5	S	32	R	24	R	0.13	S
72	LRT	R	R	S	R	R	R	R	I	R	R	S	R	1	S	32	R	12	I	0.75	S
73	Blood	R	R	R	R	R	R	R	S	R	R	R	R	0.5	S	32	R	12	I	0.38	S
74	Blood	R	R	S	R	R	R	R	I	R	R	S	R	1	S	32	R	16	R	0.5	S
75	Blood	R	R	S	R	R	R	R	S	R	R	S	R	1	S	24	R	24	R	0.5	S

76	LRT	R	R	R	R	R	R	R	S	R	R	S	R	1.5	S	32	R	32	R	0.5	S
77	LRT	R	R	S	R	R	R	R	S	R	R	S	R	0.75	S	32	R	16	R	0.25	S
78	Blood	R	R	R	R	R	R	R	S	R	R	S	R	0.75	S	32	R	12	I	0.13	S
79	Blood	R	I	R	R	R	R	S	S	R	R	S	R	1	S	32	R	16	R	0.5	S
80	Blood	R	R	R	R	R	R	R	S	R	R	S	R	2	R	32	R	24	R	0.5	S
81	Blood	R	R	R	R	R	R	R	R	R	R	S	R	1.5	S	32	R	12	I	0.75	S
82	Blood	R	R	S	R	R	R	R	S	R	R	S	R	1	S	32	R	8	I	1	S

[a]Antibiotic susceptibility results were interpreted using the European Committee on Antimicrobial Susceptibility Testing guidelines.

[b]Other includes 6 wound and 2 cerebral spinal fluid sources.

Abbreviations: I, Susceptible, increased exposure; LRT, Lower Respiratory Tract; MIC, Minimum Inhibitory Concentration; R, Resistant; S, Susceptible
